# A new species of Lonchidiidae (Hybodontiformes) from the Late Jurassic of Brazil (Aliança Formation, Jatobá Basin)

**DOI:** 10.1002/ar.70120

**Published:** 2026-01-07

**Authors:** Larissa de Souza Ribeiro, Marco Aurélio Gallo de França

**Affiliations:** ^1^ Programa de Pós‐Graduação em Ciências Biológicas e da Saúde Universidade Federal do Vale do São Francisco (UNIVASF) Petrolina Pernambuco Brazil; ^2^ Laboratório de Paleontologia e Evolução de Petrolina (LAPEP) CEMAFAUNA, UNIVASF Petrolina Pernambuco Brazil; ^3^ Colegiado de Ciências Biológicas UNIVASF Petrolina Pernambuco Brazil

**Keywords:** Hybodontiformes, Jurassic, *Parvodus*, taxonomy

## Abstract

The Aliança Formation (Jatobá Basin) represents lacustrine deposits formed in oxygenated waters that hosted a diverse fauna, including Hybodontiform sharks. Within this group, the Family Lonchidiidae comprises 11 valid genera, with *Parvodus* previously reported in Brazilian deposits from the Brejo Santo Formation (Araripe Basin, Late Jurassic) and the Aliança Formation (Jatobá Basin, Late Jurassic). Here, we describe a new species of *Parvodus* based on isolated teeth from the Aliança Formation. A taxonomic reassessment of the genus is also presented, integrating its stratigraphic and geographic distribution. The new species provides novel insights into the evolutionary history of Lonchidiidae, with implications for the paleobiogeography of the group and the paleoenvironmental interpretation of the Aliança Formation. Importantly, this record expands the known distribution of *Parvodus* into the Upper Jurassic of South America.

## 1 INTRODUCTION

Hybodontiformes are an extinct order of sharks that thrived from the Late Devonian to the end of the Cretaceous, with fossil evidence reported worldwide (Stumpf et al., 2022). During the Triassic and Jurassic, they diversified into freshwater environments, including rivers and lakes (Wen et al., 2022). Despite more than two centuries of research, knowledge of Late Jurassic Hybodontiformes remains limited, resulting in ambiguities and a poor understanding of their distribution and diversity patterns (Stumpf et al., 2022).

The family Lonchidiidae, which included sharks reaching up to ~25 cm in length (Ginter et al., 2010; Klug et al., 2010), comprises about 10 genera, such as *Jiaodontus*, *Gansuselache*, *Hylaeobatis*, *Dabasacanthus*, *Isanodus*, *Lonchidion*, *Lissodus*, *Vectiselachos*, *Parvodus*, and *Luopingselache* (Bhat et al., 2018; Wen et al., 2023). The taxonomy of Lonchidiidae has long been debated, as evolutionary and systematic analyses of this group are variable, controversial, and marked by significant gaps (see Ansorge, 1990; Duffin, 1985; Ginter et al., 2010; Rees & Underwood, 2002, 2008).

In South America, confirmed records of Lonchidiidae are restricted to *Parvodus* sp. teeth from Brazil (Cupello et al., 2012; Silva et al., 2013) and *Lonchidion paramilloensis* from Argentina (Johns et al., 2014). The genus *Parvodus* has a broad stratigraphic range, spanning from the Olenekian (Early Triassic) to the Valanginian (Early Cretaceous) (Figure 1) and was established based on material previously referred to *Lissodus rugianus* Ansorge (1990), from the Early Cretaceous of Rügen, Germany, and currently includes 10 valid species (Figure 1). These species show high levels of endemism and diverse ecological preferences, inhabiting marine, estuarine, brackish, and freshwater environments (Ansorge, 1990; Duffin, 1985; Duffin & Thies, 1997; Klug et al., 2010; Kriwet, 2004; Patterson, 1996; Rees & Underwood, 2002, 2005, 2008; Underwood & Rees, 2002; Wen et al., 2022).

**FIGURE 1:**
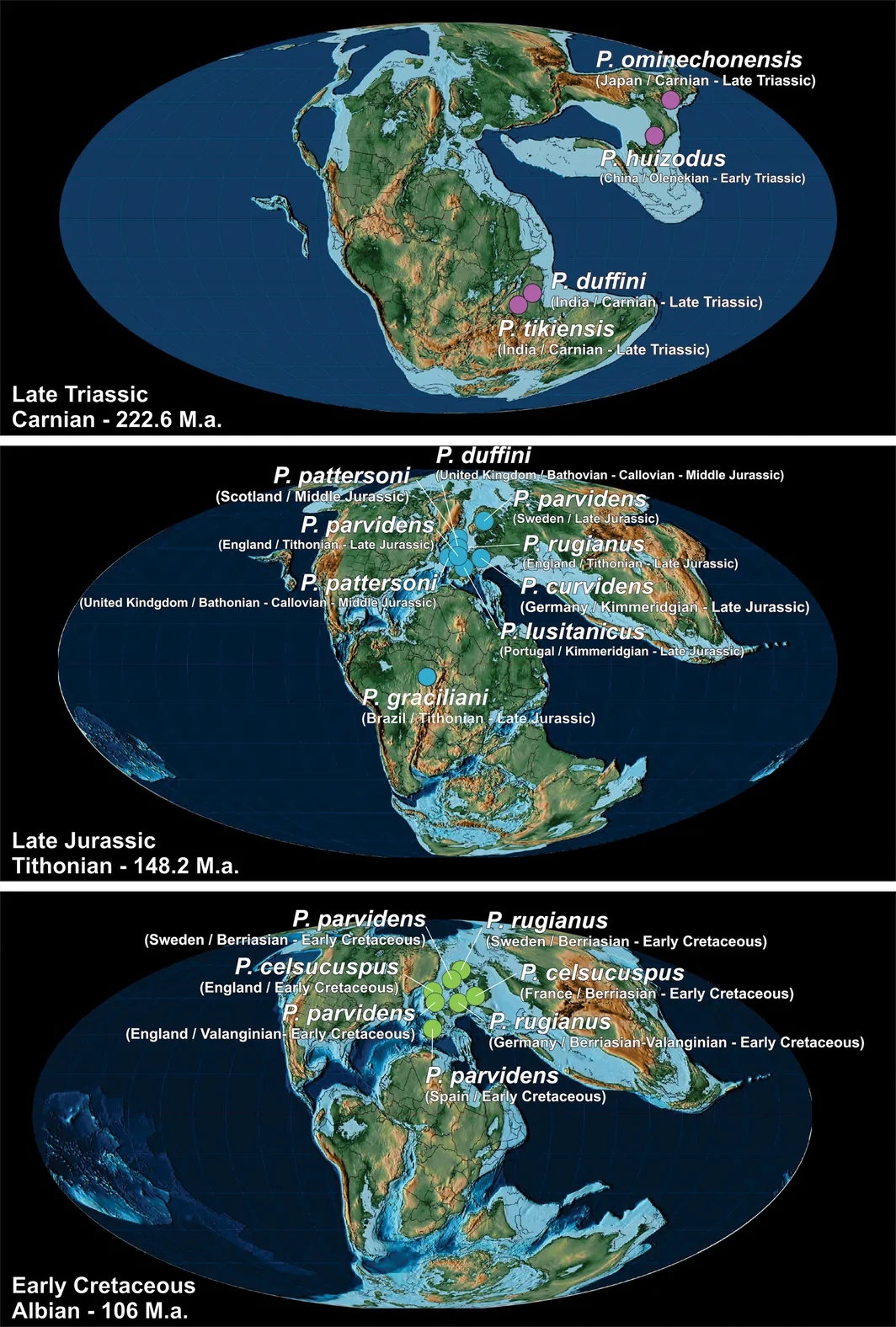
Distribution of *Parvodus* species from the Late Triassic to the Early Cretaceous. Figure modified from Scotese (2021).

Hybodontiforms reached their peak diversity during the Triassic and Jurassic, occupying a wide range of ecological niches. The Jurassic was marked by significant paleoenvironmental changes such as sea‐level fluctuations, climatic shifts, and tectonic activity that directly influenced coastal, marginal, and fluvial habitats. These changes are reflected in dental morphology, indicating varied feeding strategies and dentition types. Consequently, the study of isolated teeth is crucial for faunal and paleoenvironmental reconstructions, making the Jurassic a key period for understanding the diversity and evolutionary relationships of this order (Klug et al., 2010; Kumar et al., 2022; Leuzinger et al., 2017; Stumpf et al., 2021, 2022).

Here, we describe a new species of *Parvodus* from the Late Jurassic (Tithonian Stage) of the Aliança Formation, Jatobá Basin, in the municipalities of Ibimirim, Floresta, and Tacaratu (Pernambuco State, northeastern Brazil). In addition, we conducted a cladistic analysis and propose a phylogenetic hypothesis for the genus.

## 2 GEOLOGICAL SETTING

The specimens studied here were collected in the strata of the Aliança Formation (Jatobá Basin) at the fossil locality known as Mundubim, in the municipality of Ibimirim (State of Pernambuco), as well as from the municipality of Floresta/PE and Tacaratu/PE (Figure 2).

**FIGURE 2:**
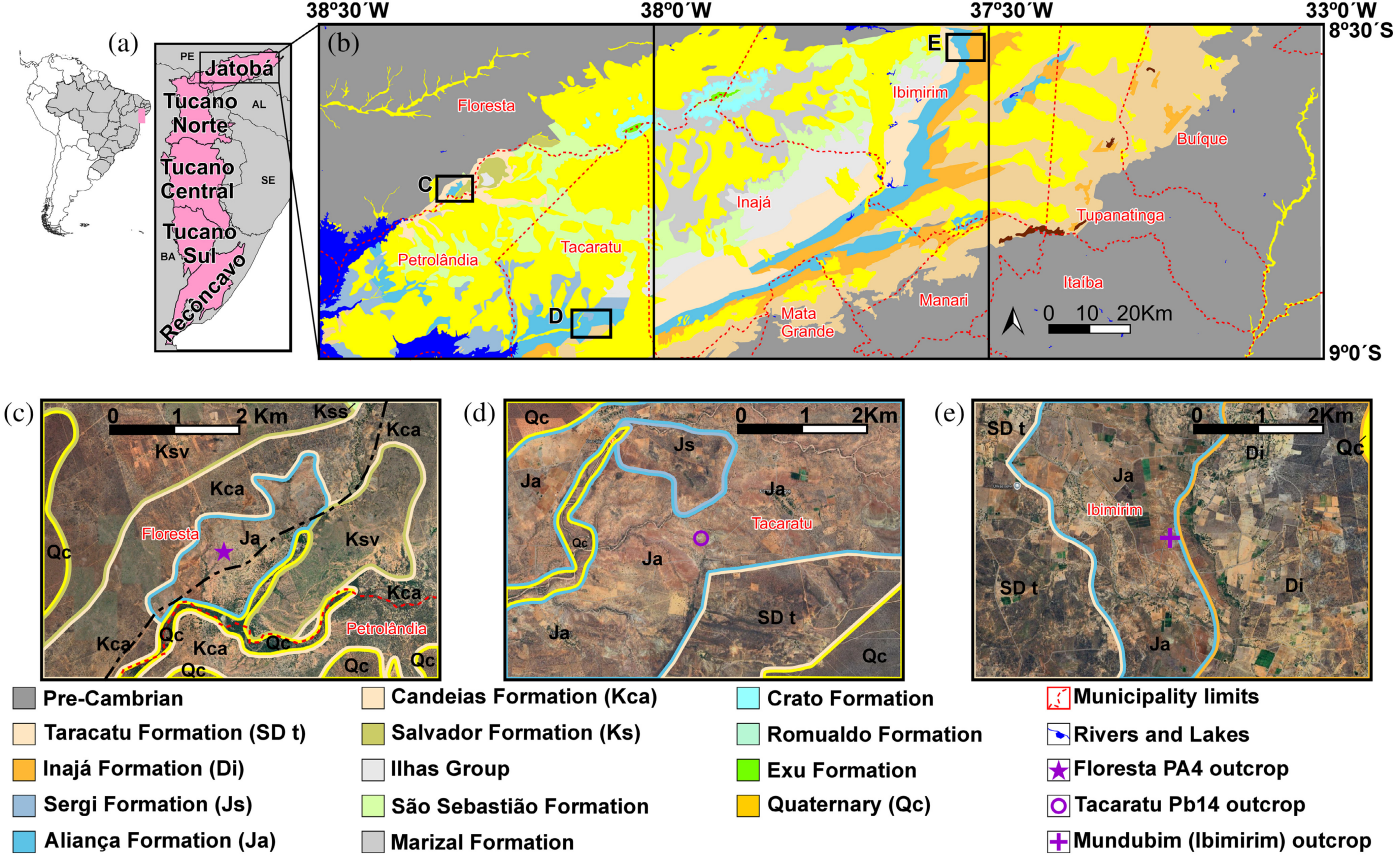
Geological map of the Jatobá Basin. (a) The Recôncavo‐Tucano‐Jatobá Rift System (RTJ) in the context of northeastern Brazil showing its associated sedimentary basins; (b) General view of the collection sites and outcrops; (c–e) Enlarged view of the collection sites: The star symbol (c) indicates the outcrop in the municipality of Floresta/PE (point PA4); the circle (d) marks the outcrop in the municipality of Tacaratu/PE (point PB14); and the cross symbol (e) represents the Mundubim outcrop in Ibimirim/PE.

The Jatobá Basin covers an area of approximately 5000 km^2^, with a NE–SW orientation. It is situated in northeastern Brazil, within the physiographic zone of the Sertão do São Francisco, extending across the central‐southern portion of Pernambuco and a small northern portion of Alagoas, in the Borborema Province. The basin is bounded to the north by the Pernambuco Lineament (de Melo Mendes et al., 2020; Silva et al., 2011). The Ibimirim Fault, which controlled the subsidence of the Jatobá Basin, is part of the Recôncavo‐Tucano‐Jatobá Rift System (RTJ), extending from Salvador/BA to Buíque/PE. The stratigraphic evolution of this basin, correlated with other basins of the RTJ, reflects the aborted development of the rift system during the Eoaptian, which is related to the breakup of South America and Africa and the subsequent opening of the South Atlantic Ocean (de Melo Mendes et al., 2020; Silva et al., 2011).

The Aliança Formation crops out in the inner eastern portion of the Jatobá Basin, ranging from north‐northeast (NNE) to extreme West‐Southwest (WSW) and is attributed to the Late Jurassic. It represents the first lacustrine phase of the basin (Pre‐Rift Tectonosequence) and is associated with the Capianga Member, characterized by red lacustrine claystones and shales, related to a regional maximum flooding surface in the Afro‐Brazilian Depression. This flooding event is mainly recorded in the RTJ Rift System and is referred to as “Capianga Lake.” The Boipeba Member, in turn, is composed of fluvial–eolian sandstones (de Melo Mendes et al., 2020; De Sousa et al., 2024; Fambrini et al., 2023). Geochemical, geological, and lithological characteristics—including whitish, green, and reddish calciferous claystones, siltstones with gypsum levels, and fossil records such as non‐marine ostracods—indicate a permanent lacustrine environment with fresh and alkaline water. This interpretation is further supported by the absence of volumetrically significant evaporite deposits. The presence of gypsum suggests episodes of evaporation in this paleolake (Capianga Lake), providing evidence for a dry paleoclimate (Gallo et al., 2010; Guzmán et al., 2015; Guzmán‐González et al., 2020; Silva, 2012).

The Capianga Member preserves a diverse Jurassic fauna, including Crocodylomorph vertebrae, teeth, and osteoderms; fish dental plates, bone fragments, and scales; as well as Hybodontiform teeth and dorsal fin spines attributable to *Planohybodus* (Hybodontidae) and Lonchidiidae. However, organic matter is absent due to oxidizing conditions and intense reworking in marginal settings, resulting in isolated and fragmented remains (De Oliveira et al., 2025).

The Aliança Formation (Jatobá Basin), traditionally assigned to the Late Jurassic, is noteworthy for its rich fossil content (including teeth, bones, and scales), which has provided valuable information for paleoenvironmental and faunal interpretations in northeastern Brazil. In this context, the unit serves as a relevant stratigraphic reference for studies of this interval (Guzmán et al., 2015; Silva, 2012).

## 3 MATERIALS AND METHODS

### 3.1 Material

The studied material comprises 24 Lonchidiidae teeth, lacking preserved roots but including specimens in both lingual and labial views, as well as anterior and posterior teeth, all exhibiting diagnostic characters relevant for taxonomic identification (Figure 3).

**FIGURE 3:**
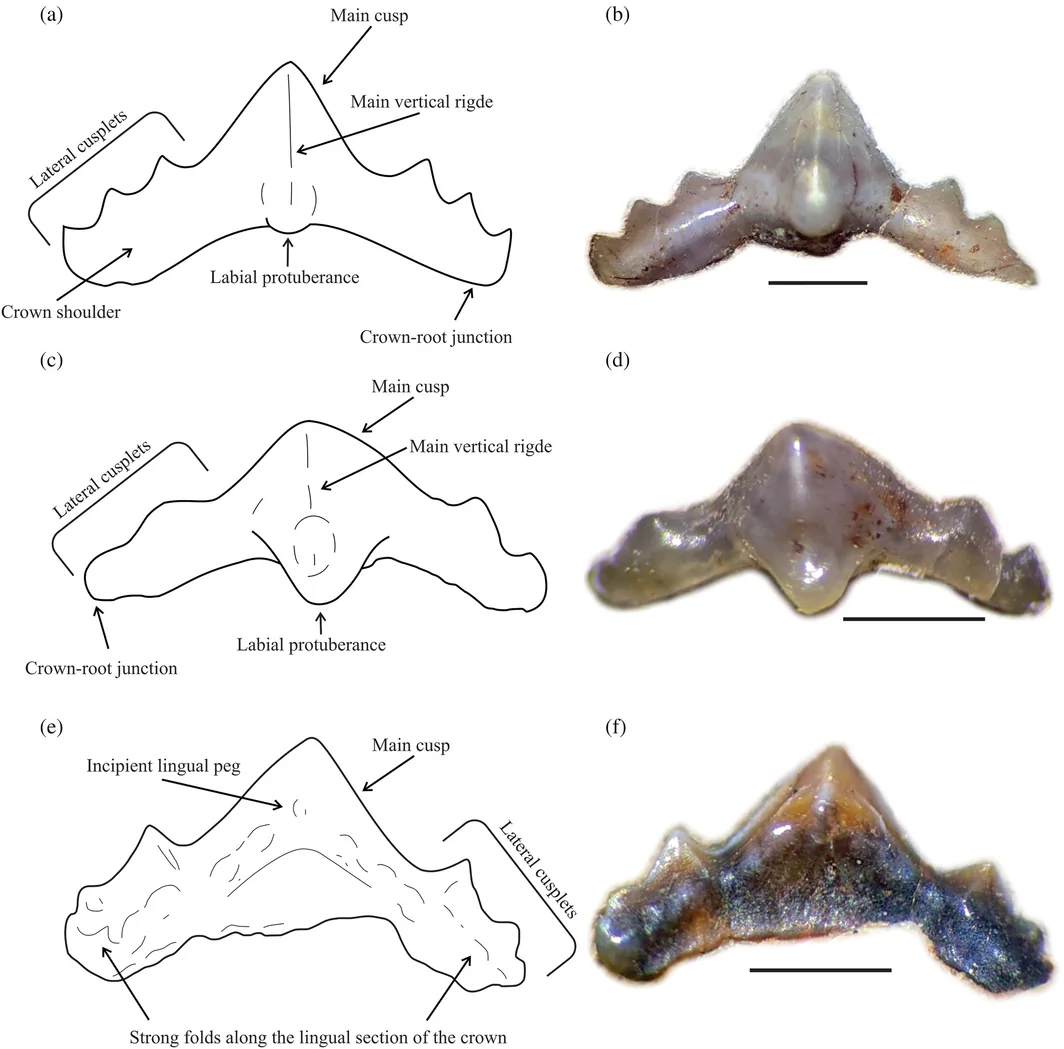
Schematic of an anterior and posterior tooth of the new species of *Parvodus*. The shapes indicate structures of taxonomic importance at the time of morphological analysis. (a) Holotype LAPEP‐0532D; (b) LAPEP‐0576C; (c) LAPEP‐0580A. Scale bar: 1 mm.

### 3.2 Preparation

All teeth analyzed in this study were mechanically prepared using needles, tweezers, glue, and brushes to carefully remove the surrounding sediment and expose the fossils, aiming to maximize the visibility of diagnostic morphological characters. The teeth of Lonchidiidae are very small and fragile, ranging from 1 to 4 mm in length and height. For this reason, a binocular stereomicroscope was used during preparation and examination of the specimens. A digital caliper was employed to take precise measurements of those specimens for which this was feasible.

### 3.3 Cladistics

The cladistic framework was established through the construction of a character matrix in Mesquite v.2.1 (Maddison & Maddison, 2007). All characters employed in the analysis were treated as equally weighted and unordered and were coded on the basis of descriptions for the 11 taxa included (see Supplementary Materials 1 and 3). The resulting dataset was subjected to a parsimony‐based phylogenetic analysis in TNT v.1.5 (Goloboff & Catalano, 2016), using the implicit enumeration algorithm to perform an exact search (see Supplementary Materials 3 and 4). The analytical procedures, including character selection, coding, and search strategy, are fully documented and therefore allow complete reproducibility of the results. This analysis should be regarded as a preliminary attempt to reconstruct the phylogeny of the group, acknowledging that future taxonomic revisions may alter the recovered relationships.

### 3.4 Note on excluded taxa

*Parvodus heterodon* has long been the subject of study and has frequently been reassigned to different genera and families. This taxon was excluded from the cladistic analysis due to discrepancies in its attribution to *Parvodus*. Its morphological features are inconsistent with the original generic diagnosis proposed by Rees and Underwood (2002), and its inclusion produced conflicting signals within the matrix, preventing a reliable referral to *Parvodus*.

By contrast, *Parvodus tikiensis*, originally assigned to *Parvodus* but transferred to the new genus *Pristrisodus tikiensis* by Bhat et al. (2018), was considered here following the revision of Wen et al. (2022). Unlike *P. heterodon*, *P. tikiensis* exhibits all diagnostic features supporting its placement within *Parvodus*.

Repositories and Institutional Abbreviations—All specimens are deposited in the Laboratório de Paleontologia e Evolução de Petrolina (LAPEP), Universidade Federal do Vale do São Francisco (UNIVASF).

## 4 SYSTEMATIC PALEONTOLOGY

Class: Chondrichthyes Huxley, 1880.

Subclass: Elasmobranchii Bonaparte, 1838.

Order: Hybodontiformes Maisey, 1975.

Superfamily: Hybodontoidea Owen, 1846.

Family: Family Lonchidiidae Herman, 1977, sensu Rees, 2008.

Genus *Parvodus* Rees & Underwood, 2002.

### 4.1 Type species

*Parvodus rugianus* (Ansorge, 1990), Lower Cretaceous (Berriasian–Valanginian) of Rügen, Germany.

### 4.2 *Parvodus graciliani* sp. nov.

Derivation of the name—The specific epithet *graciliani* honors the Brazilian writer Graciliano Ramos (1892–1953), author of the novel Vidas Secas (Ramos, 1938). This work, which portrays the lives of northeastern Brazilians, has become a symbol of human resilience in the face of the adversities of the Sertão (backlands). The name also alludes to the paleobiogeographic distribution of the genus *Parvodus*, whose species are known from Europe and Asia. This discovery represents the first recorded occurrence in South America and the first formally species proposed for Gondwana. Thus, the epithet figuratively evokes the notion of a fossil one that traversed great distances through geological time before being found in the ancient environments of northeastern Brazil.

### 4.3 Type material

Holotype LAPEP‐0532D, a nearly complete anterior tooth, lacking the root (Figures 3a,b, 4a,b, and 5a,b).

**FIGURE 4:**
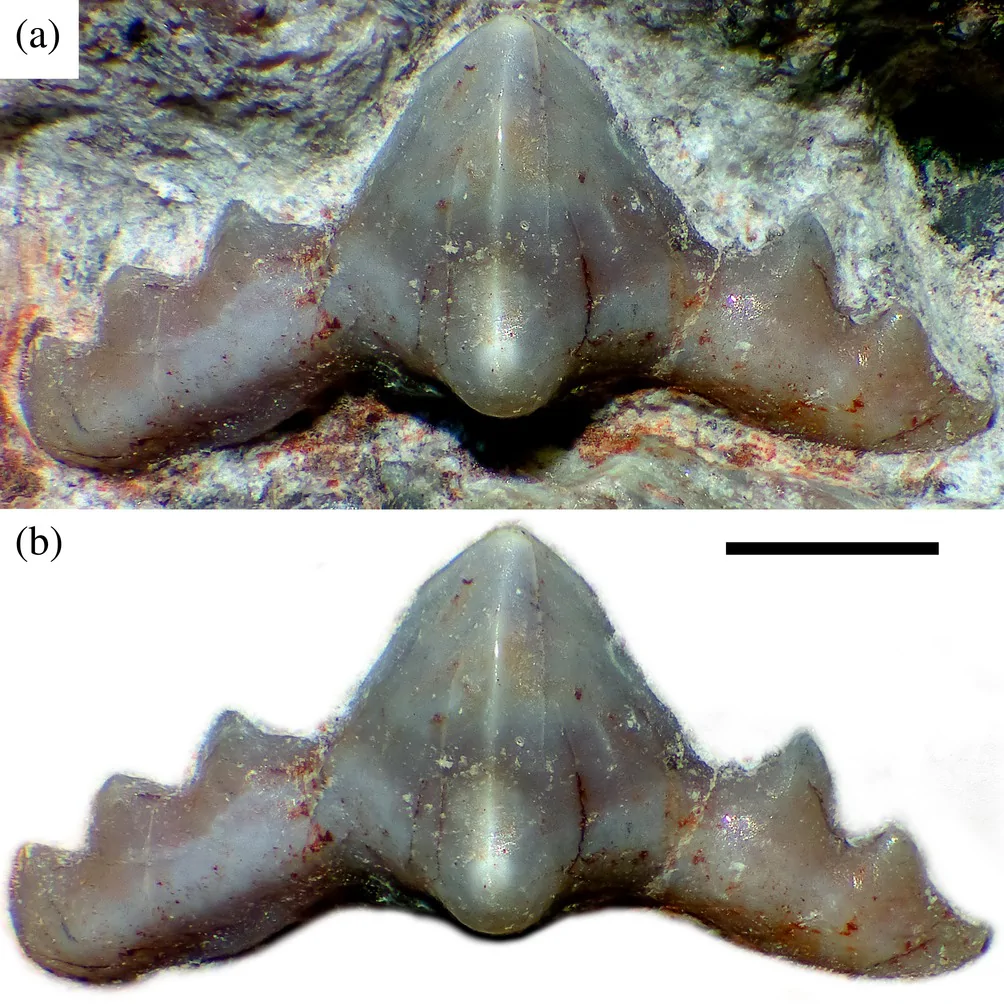
Holotype LAPEP‐0532D. A nearly complete anterior tooth of *Parvodus graciliani* sp. nov. Scale bar: 1 mm.

**FIGURE 5:**
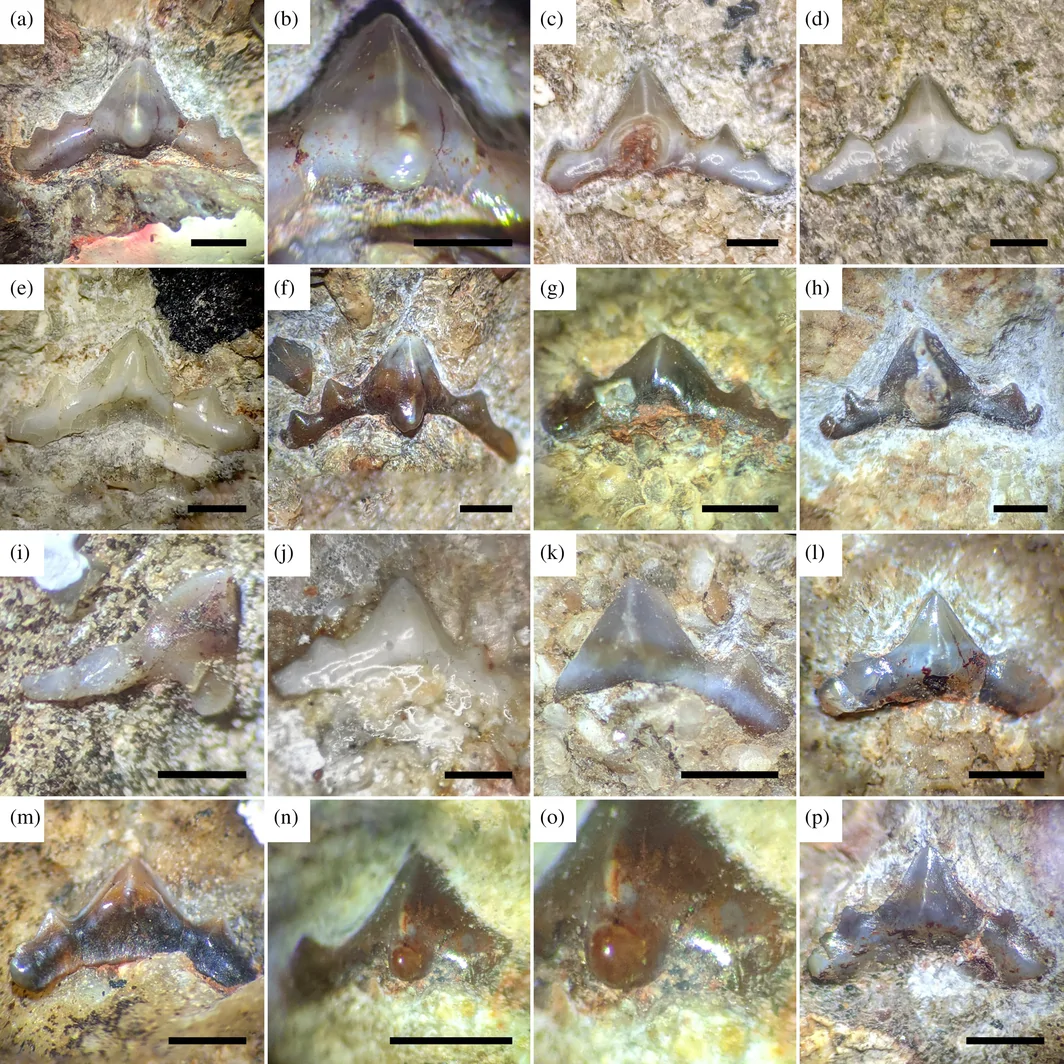
Anterior and anterolateral teeth of *Parvodus graciliani* sp. nov. (a, b), LAPEP‐0532D (Holotype), anterior tooth in labial view; (c) 0616A, anterior tooth in labial view; (d) 0619A, anterior tooth in labial view; (e) 0615A, anterior in lingual view; (f) 0575B, anterior tooth in lingual view; (g) 0543C, possibly anterior tooth in lingual view; (h) 0575C, anterior tooth in labial view (fragmented labial protuberance); (i) 0585C, anterior tooth in labial view (fragmented labial protuberance); (j) 0620A, anterolateral tooth in lingual view; (k) 0576D, anterolateral tooth in lingual view; (l) 0564B, anterolateral tooth in lingual view; (m) 0580A, anterolateral tooth in lingual view; (n, o) 0582E, anterolateral tooth in lingual view; (p) 0585B, anterolateral tooth in lingual view. Scale bar: 1 mm.

### 4.4 Type locality

The holotype was collected at the “Mundubim” outcrop, Aliança Formation (Late Jurassic), Jatobá Basin (Figure 2).

### 4.5 Material

Ibimirim/PE (“Mundubim” outcrop): LAPEP‐0532D (Holotype, anterior tooth); LAPEP‐0532C (posterior tooth); LAPEP‐0540B (posterior); LAPEP‐0543C (probably an anterolateral tooth); LAPEP‐0556A (posterior isolated tooth); LAPEP‐0556B (posterior); LAPEP‐0564B (anterolateral); LAPEP‐0566A (posterior); LAPEP‐0575B (anterior); LAPEP‐0575C (anterior); LAPEP‐0575D (posterior); LAPEP‐0576C (posterior); LAPEP‐0576D (anterolateral); LAPEP‐0580A (anterolateral); LAPEP‐0582C (posterior); LAPEP‐0582E (anterolateral); LAPEP‐0585B (anterolateral); LAPEP‐0585C (anterior) (see Supplementary Material 2).

Floresta/PE:

PA4: LAPEP‐0615A (anterior); LAPEP‐0616A (anterior); LAPEP‐0617 (posterior); LAPEP‐0619A (anterior); LAPEP‐0620A (anterolateral).

Tacaratu/PE:

PB14: LAPEP‐0618A (posterior) (see Supplementary Material 2).

### 4.6 Diagnosis

*Parvodus graciliani* sp. nov. is distinguished by the following combination of characters (synapomorphies of *Parvodus* denoted by a cross [†]; autapomorphic states of *P. graciliani* sp. nov. denoted by an asterisk [*]): (1) teeth with a rounded labial protuberance extending beyond the crown margin and reaching the crown‐root junction (†); (2) smooth labial surface of the crown, with a single vertical ridge extending from the apex of the main cusp to the central portion of the cusp (*), and terminating at the labial protuberance; (3) some teeth bear a “hook”‐shaped projection on the distal portion of the protuberance (*); (4) lingual surface of the main cusp lacking a protuberance (†); (5) lingual surface ornamented with horizontal folds on the crown shoulder of the crown, reaching the lateral cusplets (†).

### 4.7 Description

All specimens of *P. graciliani* sp. nov. are incomplete, missing their roots. The holotype, LAPEP‐0532D (Figures 4a,b and 5a,b), is better preserved than the paratypes: it shows fewer weathering artifacts that could be mistaken for taxonomically important characters, with labial protuberance not fragmented, in addition to presenting a hook‐shaped structure on its apicolabial edge of protuberance. The hook‐shaped structure is associated with the upper and lower jaw positions. Morphologically, it exhibits an elliptical outline with a pointed apical region. Lateral cusplets are well preserved, as are their height and shape of the cusplets and crown face. The holotype crown measures 1.79 mm in height and 1.37 mm in length, with only the labial face exposed. Two pairs of triangular lateral cusplets, ~0.53 mm in height, do not exceed half the height of the main cusp. A third cusplet forms the distal end of the crown on one side, while its counterpart remains embedded in the sediment, making preparation difficult due to its small size. The lateral carinae form an angle of approximately 30°, with triangular shape terminating in an incisor‐like apex. The labial crown surface is smooth, lacking folds, nodules, and ridges, but bears a single well‐defined vertical labial ridge in apicobasal orientation, extending to the central region of the labial protuberance. The rounded‐shaped protuberance is moderately developed, projecting beyond the crown‐root junction, and bears a poorly developed hook‐shaped structure distally.

### 4.8 Remarks

The smallest posterior tooth, LAPEP‐0540B (Figure 6a), measures 0.69 mm in height from the main cusp, whereas the largest posterior tooth, LAPEP‐0566A (Figure 6f), measures 1.34 and 1.04 mm in height, respectively. Among anterior teeth, the largest specimen is LAPEP‐0616A (Figure 5c), with 1.93 mm in height, while the smallest anterior tooth is 1.56 mm. Anterolateral teeth exhibit a pointed main cusp and moderate height, being considered higher than the posterior teeth but slightly lower than the anterior ones (Figure 5j–p).

**FIGURE 6:**
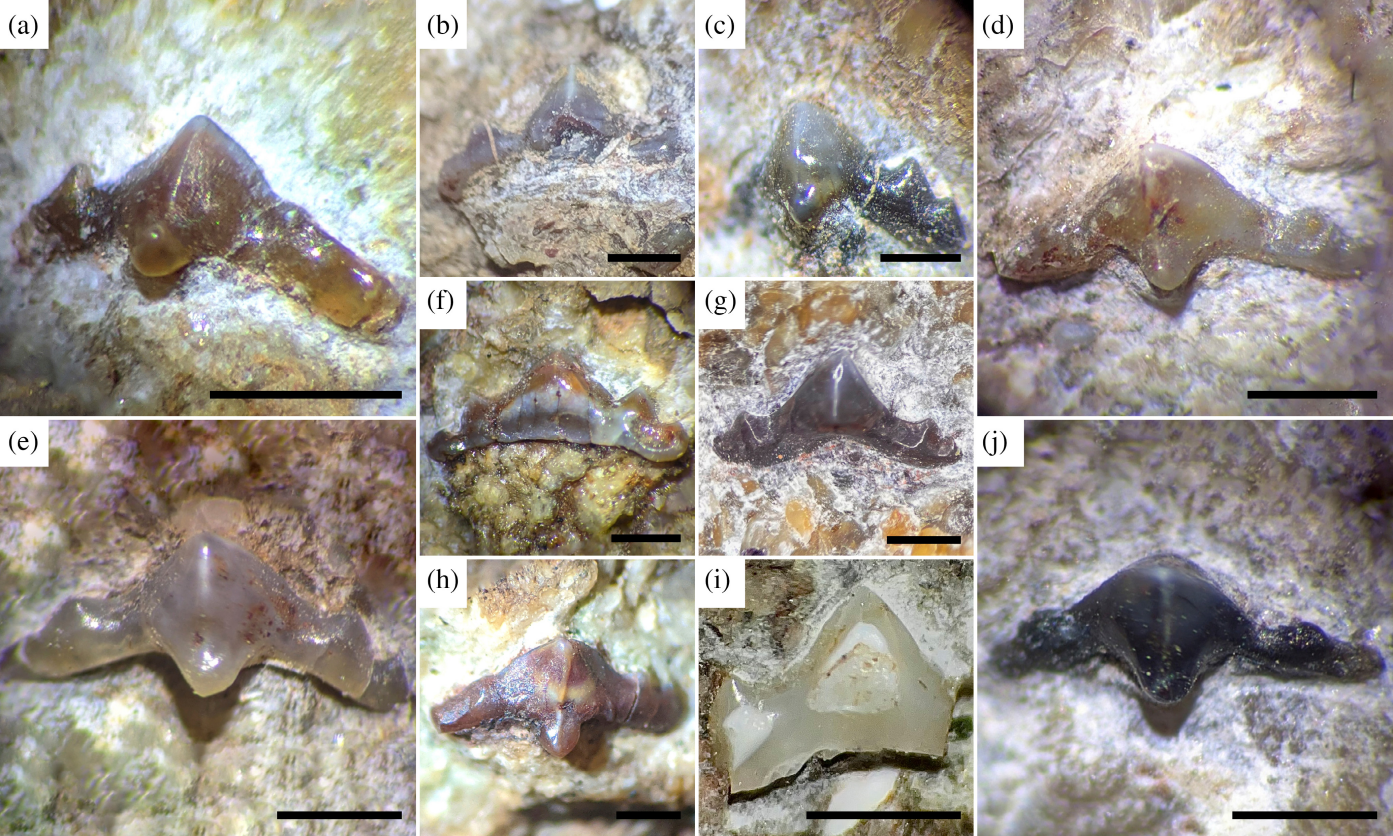
Posterior teeth of *Parvodus graciliani* sp. nov. (a), LAPEP‐0540B in labial view; (b) 0532C in labial view (fragmented labial protuberance); (c) 0575D in labial view; (d) 0556B in labial view; (e) 0576C in labial view; (f) 0566A in lingual view; (g) 0618A in lingual view; (h) 0582C, posterior tooth in labial view; (i) 0617, posterior tooth in lingual view (fragmented crown); (j) 0556A, posterior tooth in labial view; Scale bar: 1 mm.

Specimens from all outcrops bear a rounded labial protuberance. In LAPEP‐0540B (Figure 6a), it takes the form of a larger, subcircular prominence, whereas in LAPEP‐0532D (Figure 5a,b) it is less developed and more circular. A hook‐shaped projection on the distal margin of the protuberance is prominent in LAPEP‐0540B (Figure 6a), smaller in LAPEP‐0532D (Figure 5b), and broader in LAPEP‐0582E (Figure 5n,o). In LAPEP‐0575B (Figure 5f), the protuberance is distinctly rounded, projecting beyond the basal crown margin, with a slight oval excavation on its apical surface. This excavation also appears in LAPEP‐0576C and LAPEP‐0556A (Figure 6e,j), possibly reflecting in vivo wear.

Anterior teeth possess two to three pairs of cusplets, whereas posterior teeth bear only two pairs. These cusplets lack labial nodules, and the labial crown surface consistently exhibits a single vertical ridge extending from the apex of the main cusp toward the crown base or even onto the protuberance. There are no branches of this ridge, which is thin but clearly visible (Figure 3a–d). On the lingual surface, ornamentation is strong, with numerous folds ranging from moderate to pronounced, extending to the cusp bases (see Figure 5m). This feature is particularly evident in LAPEP‐0580A, 0564B, 0543C, 0585B and 0618A (Figures 5g,l,m,p and 6g), where folds occur on the crown shoulder or crown‐root junction. Some specimens, such as LAPEP‐0615A (Figure 5e), show slight lingual folds and grooves, while others, such as LAPEP‐0585B (Figure 5p), display them more subtly. In some teeth, more than one vertical ridge is present on the lingual face of the main cusp, as in LAPEP‐0564B (Figure 5l).

The dentition of *P. graciliani* sp. nov. exhibits gradual heterodonty: anterior teeth have higher crowns with sharper main cusps (Figure 3), whereas posterior teeth are lower with more rounded main cusps.

Specimens from Floresta/PE and Tacaratu/PE are indistinguishable from those of Ibimirim/PE. However, most teeth from Floresta and Tacaratu are preserved in lingual view, *P. graciliani* sp. nov. to those from Ibimirim, which are mostly preserved in labial view. Consequently, the Floresta and Tacaratu material strengthen our interpretation by providing complementary lingual‐view data for the species.

## 5 RESULTS AND DISCUSSION

### 5.1 Comparasion

According to the original diagnosis proposed by Rees and Underwood (2002), the genus *Parvodus* is defined by: (i) monognathic heterodonty; (ii) a well‐developed labial protuberance on both anterior and posterior teeth; (iii) a strong main cusp on the anterior teeth; and (iv) presence of two to three pairs of lateral cusplets. The observation of all these characters in the new species supports its inclusion within the genus.

Each described species of *Parvodus* exhibits additional diagnostic features that distinguish it from the new species (*P. graciliani* sp. nov.). In most species, crown ornamentation is extensive. *Parvodus curvidens* is the only species in which the crown bears a single, isolated vertical ridge on the main cusp (Duffin & Thies, 1997), whereas other species display denser ornamentation, with numerous pronounced horizontal and vertical ridges and, in some cases, labial nodules.

Although *P. curvidens* shares with *P. graciliani* sp. nov. the feature of a vertical ridge on the main cusp, it differs in possessing an additional vertical ridge on each lateral cusplet and in having strong labial nodules at their bases. These features are absent in the teeth analyzed here. Furthermore, *P. curvidens* has a lower crown, with a shorter main cusp, as well as longitudinal ridges on the crown shoulders and a weaker labial protuberance compared with *P. graciliani* sp. nov.

Juvenile teeth of *Parvodus pattersoni*, as described by Rees and Underwood (2008), also show an isolated vertical ridge on the main cusp, but they additionally bear labial nodules and smaller vertical ridges on the lateral cusplets. In general, the teeth of *P. pattersoni* are lower. While some of its lateral cusplets are more individualized than in other species, *P. graciliani* sp. nov. exhibits an even greater degree of separation and definition of cusps, which are also proportionally larger, consistent with the taller crown.

When compared with *P. rugianus*, *Parvodus parvidens* (Woodward, 1919), *Parvodus lusitanicus* and *Parvodus celsucuspus*, *P. graciliani* sp. nov. shares the following features: higher teeth, cusps that are well defined among themselves and with a rounded and quite prominent labial protuberance. However, the crown ornamentation *P. graciliani* sp. nov. differs markedly. In the four species, the vertical ridge on the main cusp branches into multiple ridges, a feature not observed in *P. graciliani* sp. nov. In addition, their crowns bear strong folds. *P. lusitanicus* and *P. celsucuspus* also have a much less developed labial protuberance, clearly contrasting with the robust projection observed in *P. graciliani* sp. nov. (Kriwet, 2004; Rees et al., 2013).

The teeth of *Parvodus duffini* range from high to low teeth. There are specimens described in the literature where it is possible to observe teeth with a smoother crown (Kumar & Sharma, 2013), resembling those of *P. graciliani* sp. nov., while others have more heavily ornamented crowns, differing from the new species (Rees & Underwood, 2008).

When compared with more recently described species, such as *Parvodus huizodus* (Wen et al., 2022) and *Parvodus ominechonensis* (Breeden III et al., 2024), it becomes evident that these taxa exhibit anterior teeth with a higher crown and numerous vertical ridges on the crown surface. Moreover, in some specimens of both species, the apex of the main cusp is blunt and more rounded—a condition not observed in *P. graciliani* sp. nov.

*P. tikiensis* has its morphology divided into several morphotypes, where some specimens resemble *P. graciliani* sp. nov. due to the size and shape of the labial protuberance and the shape of the main cusp, but differ from it in that the lateral cusplets are less individualized and smaller, in addition to the ornamentation of the crown having more ridges than the new species (Bhat et al., 2018; Prasad et al., 2008; Wen et al., 2022).

When comparing the material described here with previously reported *Parvodus* teeth from South America, a close similarity is observed with the specimens “UERJ‐PMB 148 a‐c” (see Cupello et al., 2012; fig. 2E,F,H,J) attributed to *Parvodus* sp. by the authors of the study. These materials described by Cupello are from the Brejo Santo Formation, Late Jurassic (Fambrini et al., 2020; Guzman‐González et al., 2016; Kuchle et al., 2011) and resemble the teeth from the new species both in height and shape of the teeth and in the shape of the more triangular main cusp with a more obtuse apical angle. Additionally, the number and delimitation of the lateral cusplets are consistent with the characteristics of the teeth studied in this work, as well as the weaker ornamentation of the crown, which is smoother, with a rounded labial protuberance, surpassing the base of the crown and containing the hook‐shaped structure in the apicolabial portion. These characteristics suggest that *P. graciliani* sp. nov. may have had a broader geographic distribution, potentially, including the Araripe Basin.

### 5.2 Cladistcs

The analysis yielded a single most parsimonious tree with 40 steps (Figures 7 and 8). *P. graciliani* sp. nov. is recovered in a polytomy together with *P. rugianus* and *P. ominechonensis*. This clade is supported by only one synapomorphy: CH 8(1) labial protuberance of main cusp on anterior teeth projecting basally to crown. However, this character is also shared convergently with *P. tikiensis* and *P. curvidens* (Figure 9a,c). This clade forms the sister group of *P. parvidens*, with which it shares the following synapomorphies: CH 12(0) size of the main cusp of the posterior teeth is higher than the width (exclusive); CH 20(1) portion between main cusp/lateral cusplets excavation and crown‐root junction in posterior teeth forming a narrow area (exclusive); and CH 14(0) depth excavation between main cusp and lateral cusplets on posterior teeth is restricted to basal half of main cusp height (convergence only with *Lonchidion humblei*).

**FIGURE 7:**
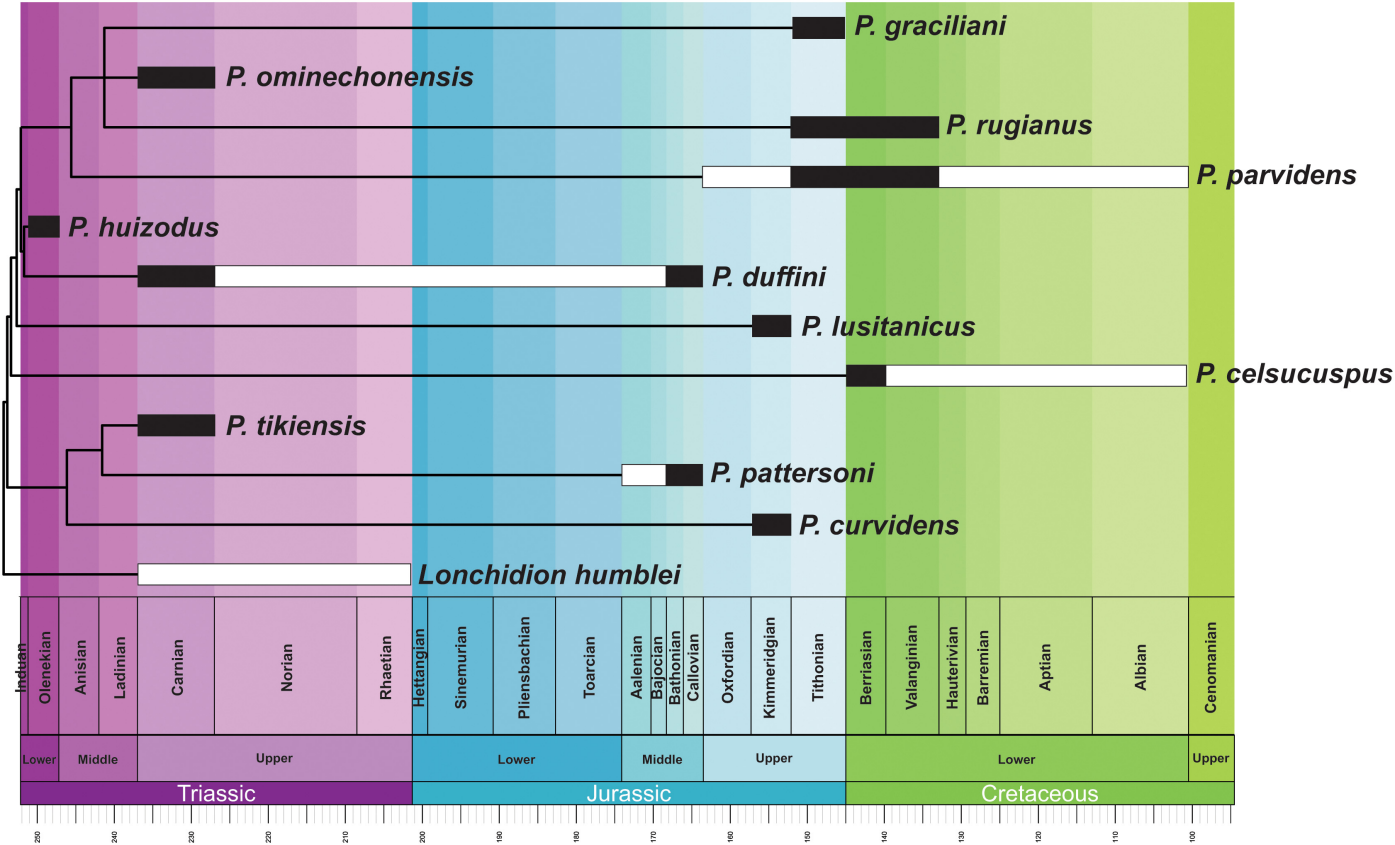
Phylogenetic relationships and stratigraphic ranges of *Parvodus* species. The phylogeny is presented within a stratigraphic framework. Black rectangles indicate occurrences constrained to specific geological periods, whereas white rectangles may represent ghost lineages between two constrained occurrences (e.g., *Parvodus duffini*) or uncertainty regarding the precise stratigraphic range, when restricted only to a broader interval (e.g., *Parvodus pattersoni*, recorded as Middle Jurassic).

**FIGURE 8:**
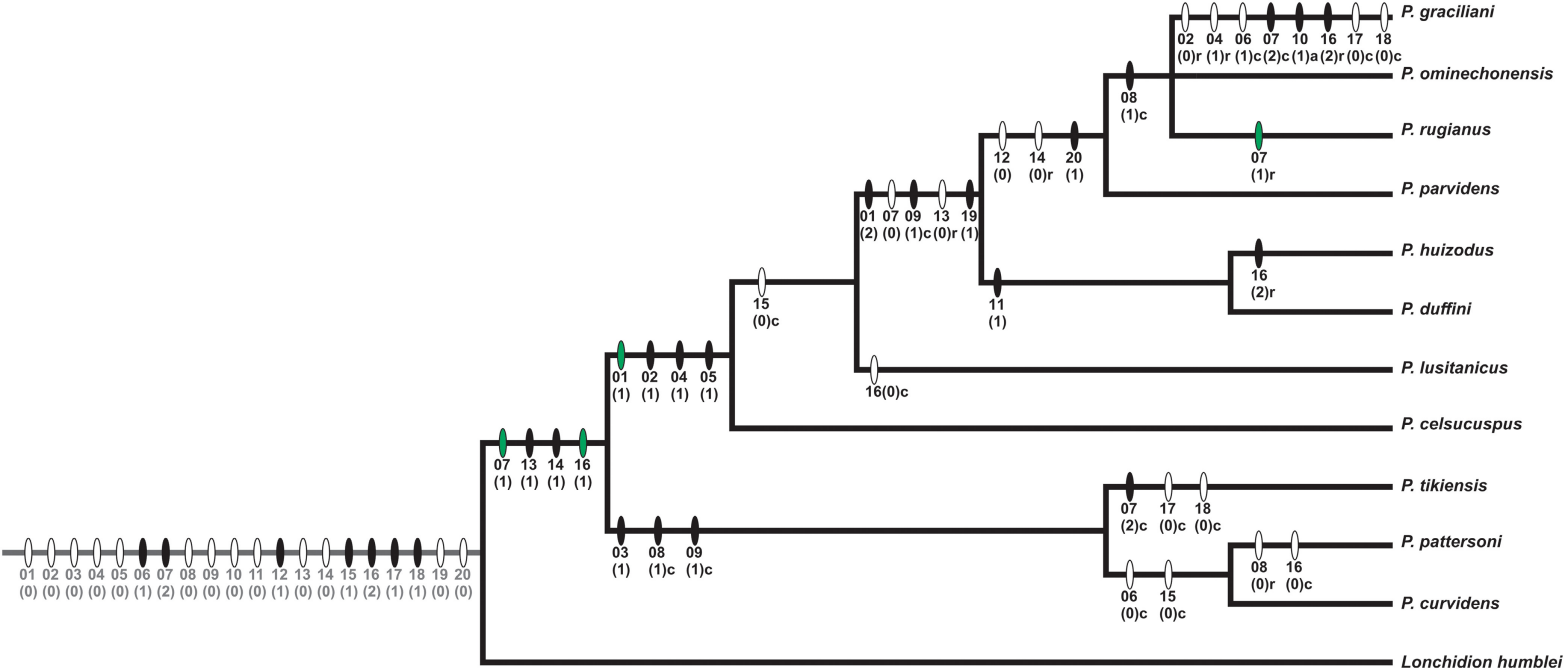
Phylogenetic hypothesis of Lonchidiidae based on 20 characters and 11 ingroup taxa, resulting in 40 steps. Colors indicate character state changes. Plesiomorphic states are represented along the most basal branch of the cladogram (in gray), with corresponding character numbers also shown in gray.

**FIGURE 9:**
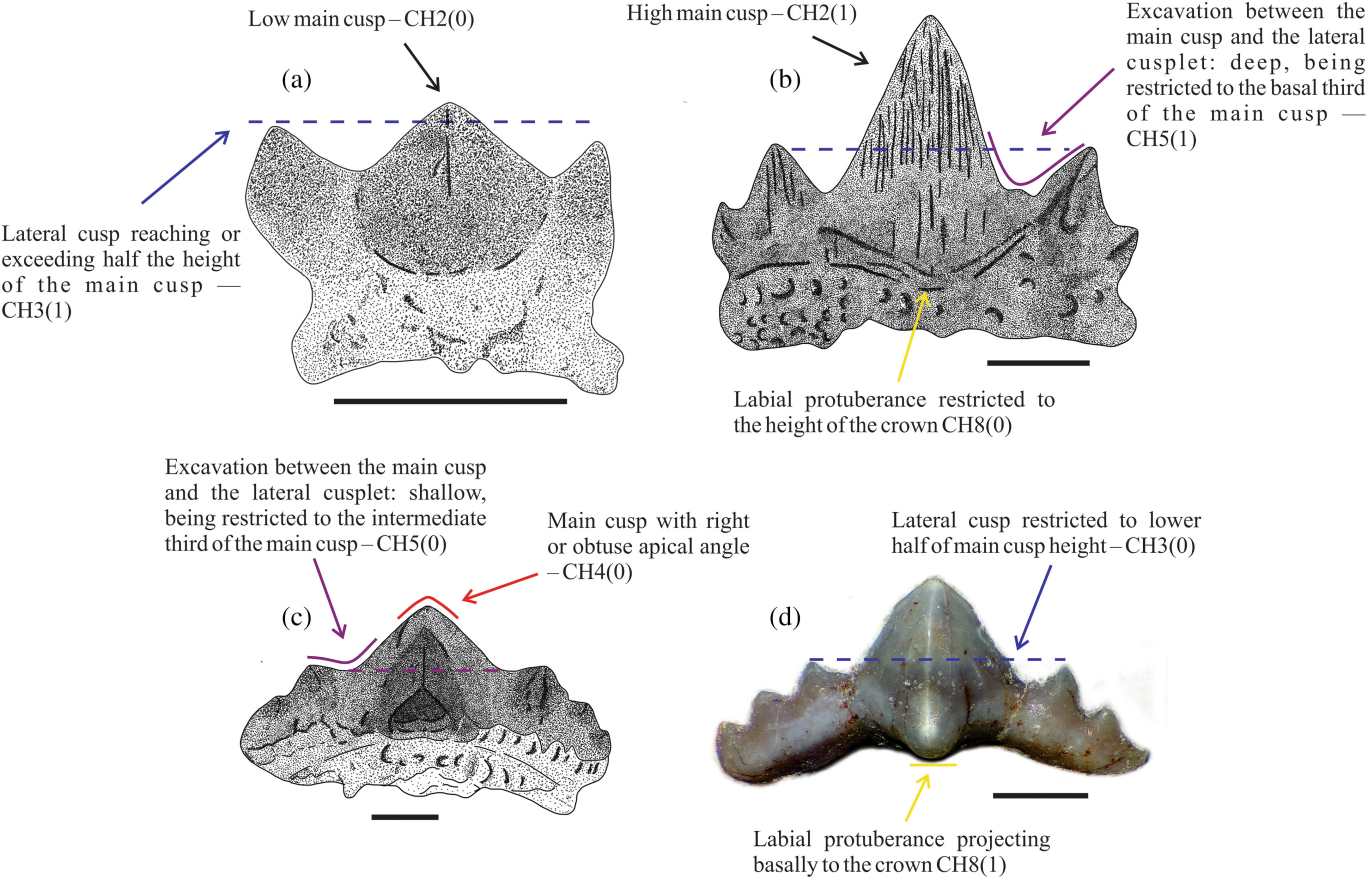
Schematic of *Parvodus* anterior teeth (A, C, D) and an anterolateral tooth (B). The shapes elucidate some characteristics that occur in other species of *Parvodus* and that contrast with those of the new species described here. (A) *Parvodus curvidens*; (B) *Parvodus lusitanicus*; (C) *Parvodus* tikiensis; (D) *Parvodus graciliani* sp. nov., Holotype LAPEP‐0532D. Scale bar: 1 mm. Adapted from Ansorge (1990); Bhat et al. (2018); Duffin and Thies (1997); Kriwet (2004); and Rees et al. (2013).

The clade comprising *P. duffini* and *P. huizodus* is supported by CH 11(1), an inclined main cusp in the anterior teeth (exclusive). This group is most closely related to the clade containing *P. parvidens* to *P. graciliani* sp. nov. This relationship is based on CH 1(2) general shape of anterior teeth is short and higher (exclusive), CH 19 (1) portion between the excavation of the main cusp/lateral cusplets and the crown‐root junction in anterior teeth is narrow area (exclusive) CH 9(1) labial protuberance of main cusp on posterior teeth projected basally to crown (convergence of *P. curvidens* + *P. tikiensis* + *P. pattersoni*) (Figures 7, 8, and 9a,c), and CH 13(0) correlation of size between lateral cusplet and main cusp on posterior teeth is restricted to the lower half of the main cusp height (only also observed in *L. humblei*).

*P. celsucuspus* and *P. lusitanicus* form successive sister clades related to the clade composed of *P. duffini* plus *P. graciliani* sp. nov. The closer position of *P. lusitanicus* (Figure 9b) is supported by a single characteristic: CH 15(0) lateral cusplets size on posterior teeth is height similar to width (also observed in *P. pattersoni* and *P. tikiensis*). The positioning of *P. celsucuspus* occurs at the early basal divergence of this branch, and this branch is very well supported by four synapomorphies: CH 1(1) shape of anterior teeth is short and higher (exclusive); CH 5(1) excavation between the main cusp and lateral cusplets on anterior teeth is deep, being restricted to the basal third of the main cusp (exclusive); CH 2(1) size of the main cusp of the anterior teeth is high, that is height is greater than width (only shared with *P. graciliani* sp. nov.); and CH 4(1) apical angle of the main cusp on anterior teeth forms an acute angle (also only shared with *P. graciliani* sp. nov.).

The three remaining taxa of the genus *Parvodus* form a group positioned at the base of the cladistic tree. This relationship is supported by the sharing of CH 3(1) lateral cusplets reaches or exceeds half of the main cusp height (exclusive), and CH 9(1) labial protuberance of main cusp on posterior teeth projected basally to crown (previously cited as convergent in the clade composed of *P. duffini* plus *P. graciliani* sp. nov.). Within this group, *P. pattersoni* and *P. tikiensis* are most closely related, supported by two synapomorphies: CH 6(0) size of lateral cusps on anterior teeth is height similar to width (convergent with *P. graciliani* sp. nov.), and CH 15(0) lateral cusplets size on posterior teeth is height similar to width (previously cited as also convergent with the clade *P. lusitanicus* + *P. graciliani* sp. nov.).

In most *Parvodus* species, the labial surface of the crown is ornamented with folds and vertical ridges on all cusps. By contrast, the teeth of *P. graciliani* sp. nov. (Figures 3a–d and 9d) bear only a single vertical ridge on the central cusp, while the lateral cusplets remain smooth. When examining *P. pattersoni*, one must consider its intraspecific variation in ornamentation since it can resemble that of the new species. *P. curvidens*, although also possessing only one vertical ridge on the main cusp, is distinguished by at least one additional ridge on each flank of that cusp.

### 5.3 Paleoecological implications

Few studies have addressed the paleoecological dynamics of Capianga Lake (Aliança Formation). The records presented here provide valuable insights, indicating a wave‐influenced freshwater environment with a distinct trophic structure.

According to paleolimnological and lithostratigraphic data available in the literature (De Sousa et al., 2024; Guzmán‐González et al., 2020), this lake experienced a brief phase of saline conditions, and its waters were likely clear and warm. Moreover, its temperature may have increased at certain intervals, contributing to elevated evaporation rates. These lines of evidence support the euryhaline tolerance of Hybodontiformes, and by extension, also suggest this condition for the genus *Parvodus*.

When analyzing the dentition of *Parvodus*, it becomes evident that the teeth exhibit a clutching‐type dentition, characterized by pointed main cusps adapted for grasping small, soft prey rather than cutting or crushing. This suggests a carnivorous diet, likely consisting of small fish, such as juvenile *Lepidotes*, and possibly invertebrates. Considering their small body size only 5–25 cm in total length (Ginter et al., 2010; Klug et al., 2010; Rees & Underwood, 2002), *Parvodus* probably occupied mid‐trophic levels within the lacustrine ecosystem. They could have competed with other Hybodontiforms, such as *Planohybodus*. At higher trophic levels, predators such as the large Sarcopterygian *Mawsonia* (4–5 m in length) and sizable Actinopterygians like *Lepidotes* and *Ceratodus* (Silva, 2012) likely fed on a variety of fishes. *Mawsonia* likely acted as one of the apex predators, preying even on *Lepidotes* and *Ceratodus*. *Crocodylomorphs*, in turn, could feed on fishes, including *Mawsonia* (De Araújo Carvalho et al., 2021), thereby regulating fish populations in the lake.

The depth of Capianga Lake has been the focus of debate in several studies. While some authors describe it as an extensive system with shallow, oxygenated, and seasonally stable waters alternating between flood and drought (De Oliveira et al., 2025; Fambrini et al., 2023; Guzmán‐González et al., 2020; Silva, 2012), others argue that its depth varied, with shallower and fresher waters in the northeastern basin compared to the southeast (De Sousa et al., 2024).

Future research on the Aliança Formation and the Capianga Member will be crucial for establishing a more consistent framework of lithostratigraphic features and faunal reconstructions, thereby improving our understanding of the paleoenvironmental dynamics and structural evolution of Capianga Lake during the Jurassic (De Sousa et al., 2024).

Finding *P. graciliani* sp. nov. in both sectores of the Lake (to the north, in the Ibimirim municipality region, and further south, in the municipalities of Floresta and Tacaratu municipality), with no observed variation in the teeth size among the preserved specimens, suggests that this taxon may have had a broad distribution, rather than being restricted to shoreline habitats or deeper‐water environments.

## 6 CONCLUDING REMARKS

Significant gaps persist in the taxonomy of the genus *Parvodus*, partly due to varying emphasis on morphological characters by different authors in studies of Lonchidiidae. Our results underscore the need for further refinement and precise description of these characters. Despite taxonomic challenges arising from the small and fragile nature of *Parvodus* specimens, the material described here confirms the genus for Brazil and represents the first record of a new *Parvodus* species from the Late Jurassic of the Aliança Formation (Jatobá Basin) and South America. This identification is supported by diagnosis of the genus, including the labial protuberance, up to three pairs of lateral cusps, and few vertical crests on the crown, as well as exclusive features of the new species described here, such as a single vertical crest on the main cusp—without additional crests on the lateral cusps—and a hook‐shaped structure at the apex of the labial protuberance, which is rounded or elliptical. This discovery not only clarifies the morphological characterization of the genus but also provides important insights into the paleobiogeography and evolutionary history of Hybodontiforms.

## AUTHOR CONTRIBUTIONS

**Larissa de Souza Ribeiro:** Conceptualization; investigation; writing – original draft; methodology; validation; visualization; writing – review and editing; software; formal analysis; data curation. **Marco Aurélio Gallo de França:** Conceptualization; funding acquisition; methodology; writing – review and editing; software; data curation; supervision; resources; formal analysis; project administration.

## Supporting information


**Data S1.** Supporting Information.
