# Supplementary material for: A new species of Lonchidiidae (Hybodontiformes) from the Late Jurassic of Brazil (Aliança Formation, Jatobá Basin)
Source: Anat Rec (Hoboken). 2026 Jan 7;309(11):3091–105. doi: 10.1002/ar.70120 (PMC13644892; doi:10.1002/ar.70120)
Supplement: Supplementary file 1 [file AR-309-3091-s001.docx]

**Supplementary material 1**

List of *Parvodus* species and their respective references where the search was carried out to encode the characters, as well as the occurrence of the species

| **Species** | **Reference** | **Temporal distribution** | **Geological Formation** | **Location (Country)** |
| --- | --- | --- | --- | --- |
| *P. rugianus* | (1) Ansorge, 1990;  (2) Underwood and Rees, 2002; (3) Rees and Underwood, 2002; | (1) Berriasian-Valanginian (Early Cretaceous); (2) Tithonian (Late Jurassic) and Valanginian (Early Cretaceous); (3) Berriasian (Early Cretaceous) | (1) Erratic deposit on the Pleistocene marl cliff of Lobber Ort, Wealden, Rügen, Mönchgut península; (2) Durlston Formation - Durlston Bay, Dorset | (1) Germany, Europe; (2) Southern England, Europe; (3) Southern Sweden, Europe |
| *P. pattersoni* | Ansorge, 1990; (1, 2) Rees and Underwood, 2005, 2008; (3, 4) Duffin, 1985; Shang, Cuny and Chen, 2008 | (1) Middle Jurassic; (2) Bathonian and Callovian (Middle Jurassic; (3, 4) Bathonian (Middle Jurassic) | (1) Lealt Shale Formation - Kildonnan Member; (2) Sharps Hill Formation; (3) Forest Marble, Watton Cliff, Dorset; (4) Great Oolite Group | (1) Isle of Eigg – Scotland, Europe; (2) Oakham and Hornsleasow Quarry - United Kingdom, Europe; (3) England, Europe; (4) Old Cement Works Quarry, Kirtlington, Oxfordshire, England, Europe |
| *P. curvidens* | Duffin and Thies, 1997 | Kimmeridgian (Late Jurassic) | Cement quarry called Rohstoftbetriebe Oker, on the south shore of the Langenberg | Northwest Germany, Europe |
| *P. lusitanicus* | Kriwet, 2004; Rees *et al*., 2013 | Kimmeridgian (Late Jurassic) | Guimarota Coal Mine, near Leira | Central Portugal, Europe |
| *P. celsucuspus* | (1) Rees *et al*., 2013; (2) Allain *et al*., 2021; (3) Penn and Sweetman, 2023 | (1) Early to Middle Berriasian (Early Cretaceous); (2) Late Berriasian (Early Cretaceous); (3) Early Cretaceous | (1) Northern margin of the Aquitaine Basin; (2) Angeac-Charente Lagerstätte. It is located in Angoulême, in the New Aquitaine region; (3) Wessex Basin, basal Wessex Formation (Wealden Group) | (1) Carrière de Champblanc Quarry in the Village of Cherves-de-Cognac - South-West France – Europe; (2) South-west France; (3) Dungy Head coastal headland, Dorset - Southern England, Europe |
| *P. huizodus* | Wen *et al*., 2022 | Olenekian (Early Triassic) | Dongchuan Formation | Near Zhaojia Village (Huize County, Yunnan Province) in southwest China, Asia |
| *P. ominechonensis* | Breenden III *et al*., 2024 | Carnian (Late Triassic) | Momonoki Formation (Mine Group) | Mine, Yamaguchi, southwest Honshu – Japan, Asia |
| *P. duffini* | (1) Kumar e Sharma, 2013; (2) Rees and Underwood, 2008 | (1) Early Carnian (Late Triassic); (2) Bathonian and Callovian (Middle Jurassic) | (1) Rewa Basin (Tiki Formation); (2) Sharps Hill Formation | (1) India, Asia; (2) Oakham and Hornsleasow Quarry - United Kingdom, Europe |
| *P. parvidens* | (1) Bermudéz-Rochas, 2009; (2) Rees, 2002; (3) Rees, 1996; (4) Blake *et al*., 2024; (5) Underwood e Rees, 2002; Oreska, Carrano and Dzikiewicz, 2013; Rees, 2002; Wen *et al*., 2022 | (1, 4) Early Cretaceous; (2) Berriasian (Early Cretaceous); (3) Late Jurassic; (5) Tithonian (Late Jurassic) and Valanginian (Early Cretaceous) | (1) Basque-Cantabrian Basin (Vega de Pas Formation, Viviparus bed Member); (2) Annero Formation (Vitabäck Clays Member); (3) Vitabäck Clays; (4) Lower Greensand Group; (5) Durlston Formation | (1) Cantabrian Mountains, Province of Catarb - northern Spain, Europe; (2) Erisdal, Southern Sweden, Europe; (3) Erisdal, Scania - Southern Sweden, Europe; (4) Clophill, Bedfordshire, UK and Nutfield, UK; (5) Durlston Bay, Dorset - Southern England, Europe |
| *P. tikiensis* | Bhat, Ray and Datta, 2018; Kumar e Sharma, 2013; Ray *et al*., 2016; Singh *et al*., 2020 | Early Carnian (Late Triassic) | Rewa Basin (Tiki Formation) | India, Asia |
| *P. gracilianus* | Presented here | Tithonian (Late Jurassic) | Aliança Formation, Jatobá Basin | Brazil, South America |
| *Lonchidion humblei* | (1)Heckert, Ivanov, Lucas, 2007; (2) Murry, 1981 | (1, 2) Late Triassic | (1) Chinle Group, Chinle Formation  (2) Dockum Group, Tecovas Formation | (1, 2) Texas - United States, North America |

**Supplementary material 2**

List of *P. gracilianus* specimens collected during this work, as well as the location of each one.

| **Specimens analyzed** | **Location (gps)** | **Municipality/Outcrop** |
| --- | --- | --- |
| LAPEP-0532D (Holotype) | 24L 0661592/9056388 | Ibimirim, PE, BR (Mudubim outcrop) |
| LAPEP-0532C | 24L 0661592/9056388 | Ibimirim, PE, BR (Mudubim outcrop) |
| LAPEP-0540B | 24L 0661592/9056388 | Ibimirim, PE, BR (Mudubim outcrop) |
| LAPEP-0543C | 24L 0661592/9056388 | Ibimirim, PE, BR (Mudubim outcrop) |
| LAPEP-0556A | 24L 0661592/9056388 | Ibimirim, PE, BR (Mudubim outcrop) |
| LAPEP-0556B | 24L 0661592/9056388 | Ibimirim, PE, BR (Mudubim outcrop) |
| LAPEP-0564B | 24L 0661592/9056388 | Ibimirim, PE, BR (Mudubim outcrop) |
| LAPEP-0566A | 24L 0661592/9056388 | Ibimirim, PE, BR (Mudubim outcrop) |
| LAPEP-0575B | 24L 0661592/9056388 | Ibimirim, PE, BR (Mudubim outcrop) |
| LAPEP-0575C | 24L 0661592/9056388 | Ibimirim, PE, BR (Mudubim outcrop) |
| LAPEP-0575D | 24L 0661592/9056388 | Ibimirim, PE, BR (Mudubim outcrop) |
| LAPEP-0576C | 24L 0661592/9056388 | Ibimirim, PE, BR (Mudubim outcrop) |
| LAPEP-0576D | 24L 0661592/9056388 | Ibimirim, PE, BR (Mudubim outcrop) |
| LAPEP-0580A | 24L 0661592/9056388 | Ibimirim, PE, BR (Mudubim outcrop) |
| LAPEP-0582C | 24L 0661592/9056388 | Ibimirim, PE, BR (Mudubim outcrop) |
| LAPEP-0582E | 24L 0661592/9056388 | Ibimirim, PE, BR (Mudubim outcrop) |
| LAPEP-0585B | 24L 0661592/9056388 | Ibimirim, PE, BR (Mudubim outcrop) |
| LAPEP-0585C | 24L 0661592/9056388 | Ibimirim, PE, BR (Mudubim outcrop) |
| LAPEP-0615A | 24L 9033006/577083 | Floresta, PE, BR (PA4) |
| LAPEP-0616 | 24L 9033006/577083 | Floresta, PE, BR (PA4) |
| LAPEP-0617 | 24L 9033006/577083 | Floresta, PE, BR (PA4) |
| LAPEP-0619A | 24L 9033006/577083 | Floresta, PE, BR (PA4) |
| LAPEP-0620 | 24L 9033006/577083 | Floresta, PE, BR (PA4) |
| LAPEP-0618A | 24L 9010881/599734 | Floresta, PE, BR (PB14) |

**Supplementary material 3**

Characters considered and analyzed for construction and coding of the Matrix.

| 1 | General Shape of anterior teeth: (0) Long and Higher (height is less than 50% of the length); (1) Short and higher (height is greater than 55% of length); (2) short and low (the height is between 50 and 55% of the length) |
| --- | --- |
| 2 | Size of the Main Cusp of the Anterior Teeth: (0) Low (height is less than or equal to width); (1) High (height is greater than width) |
| 3 | Size correlation between lateral cusplets and main cusp on Anterior Teeth: (0) Restricted to the lower half of the main cusp height; (1) reaches or exceeds half of the main cusp height |
| 4 | Apical angle of the main cusp on anterior teeth: (0) straight or obtuse angle; (1) Acute angle |
| 5 | Depth excavation between the main cusp and lateral cusplets on anterior teeth: (0) Shallow, being restricted to the intermediate third of the main cusp; (1) Deep, being restricted to the basal third of the main cusp |
| 6 | Size of lateral cusps on anterior teeth: (0) Height similar to Width; (1) Evidently higher than Width |
| 7 | Ornamentation on anterior teeth: (0) on the main cusp and lateral cusplets, with one main vertical ridge on the main cusp and several accessory vertical ridges (>5); (1) on the main cusp and lateral cusplets, with one main vertical ridge on the main cusp and few accessory vertical ridges (<4); (2) Only one vertical ridge on the main cusp, with some incipient ones (1 or 2) on the lateral cusplets |
| 8 | Labial protuberance of main cusp on anterior teeth: (0) Restricted to crown height; (1) may project basally to crown |
| 9 | Labial protuberance of main cusp on posterior teeth: (0) Restricted to crown height; (1) may project basally to crown |
| 10 | Labial protuberance with hook-shaped distal portion: (0) absent; (1) present |
| 11 | Inclination of the main cusp in the anterior teeth: (0) Not inclined or nearly perpendicular; (1) Inclined |
| 12 | Size of the Main Cusp of the Posterior Teeth: (0) Higher than the Width; (1) Height similar to or less than the Width |
| 13 | Correlation of size between lateral cusplet and main cusp on Posterior Teeth: (0) Restricted to the lower half of the main cusp height; (1) reaches or exceeds half of the main cusp height |
| 14 | Depth excavation between main cusp and lateral cusplets on posterior teeth: (0) Restricted to basal half of main cusp height; (1) Restricted to apical half of main cusp height |
| 15 | Lateral cusplets size on posterior teeth: (0) Height similar to Width; (1) Clearly higher than Width |
| 16 | Ornamentation on posterior teeth: (0) Main and accessory vertical ridges on main cusp and lateral cusplets; (1) Main vertical ridge on main cusp and lateral cusplets (there may be some incipient accessory vertical ridge); (2) only main vertical ridge on main cusp (absent on lateral cusplets) |
| 17 | Basal margin of anterior teeth: (0) laterally inclined in basal direction; (1) Almost linear (straight) |
| 18 | Basal margin of posterior teeth: (0) laterally inclined in basal direction; (1) Almost linear (straight) |
| 19 | Portion between the excavation of the main cusp/lateral cusplets and the crown-root junction in anterior teeth: (0) Elevated, forming a plateau between the junction and the cusplets; (1) Narrow area |
| 20 | Portion between main cusp/lateral cusplets excavation and crown-root junction in posterior teeth: (0) Raised, forming a plateau between the junction and the cusplets; (1) Narrow area |

**Supplementary material 4**

Matrix of the genus *Parvodus* coded. LAPEP species included and with *Lonchidion humblei* as outgroup.

| **Táxons** | **C1** | **C2** | **C3** | **C4** | **C5** | **C6** | **C7** | **C8** | **C9** | **C10** | **C11** | **C12** | **C13** | **C14** | **C15** | **C16** | **C17** | **C18** | **C19** | **C20** |
| --- | --- | --- | --- | --- | --- | --- | --- | --- | --- | --- | --- | --- | --- | --- | --- | --- | --- | --- | --- | --- |
| *Lonchidion humblei* | 0 | 0 | 0 | 0 | 0 | 1 | 2 | 0 | 0 | 0 | 0 | 1 | 0 | 0 | 1 | 2 | 1 | 1 | 0 | 0 |
| *P. curvidens* | 0 | 0 | 1 | 0 | 0 | 1 | 2 | 1 | 1 | 0 | 0 | 1 | 1 | 1 | 1 | 1 | 1 | 1 | 0 | 0 |
| *P. tikiensis* | 0 | 0 | 1 | 0 | 0 | 0 | 1 | 1 | 1 | 0 | 0 | 1 | 1 | 1 | 0 | 1 | 0 | 0 | 0 | 0 |
| *P. lusitanicus* | 1 | 1 | 0 | 1 | 1 | 1 | 0 | 0 | 0 | 0 | 0 | 1 | 1 | 1 | 0 | 0 | 1 | 1 | 0 | 0 |
| *P. celsucuspus* | 1 | 1 | 0 | 1 | 1 | 1 | 1 | 0 | 0 | 0 | 0 | 1 | 1 | 1 | 1 | 1 | 1 | 1 | 0 | 0 |
| *P. ominechonensis* | 2 | 1 | 0 | 1 | 1 | 1 | 0 | 1 | ? | 0 | 0 | ? | ? | ? | ? | ? | 1 | ? | 1 | ? |
| *P. duffini* | 2 | 1 | 0 | 1 | 1 | 1 | 0 | 0 | ? | 0 | 1 | ? | ? | ? | ? | ? | 1 | ? | 1 | ? |
| *P. pattersoni* | 0 | 0 | 1 | 0 | 0 | 0 | 1 | 0 | 1 | 0 | 0 | 1 | 1 | 1 | 0 | 0 | 1 | 1 | 0 | 0 |
| *P. huizodus* | 2 | 1 | 0 | 1 | 1 | 1 | 0 | 0 | 1 | 0 | 1 | 1 | 0 | 1 | 0 | 2 | 1 | 1 | 1 | 0 |
| *P. parvidens* | 2 | 1 | 0 | 1 | 1 | 1 | 0 | 0 | 1 | 0 | 0 | 0 | 0 | 0 | 0 | 1 | 1 | 1 | 1 | 1 |
| *P. rugianus* | 2 | 1 | 0 | 1 | 1 | 1 | 1 | 1 | 1 | 0 | 0 | 0 | 0 | 0 | 0 | 1 | 1 | 1 | 1 | 1 |
| *P. gracilianus* | 2 | 0 | 0 | 0 | 1 | 0 | 2 | 1 | 1 | 1 | 0 | 0 | 0 | 0 | 0 | 2 | 0 | 0 | 1 | 1 |

**References**

ALLAIN, R. *et al*. Vertebrate paleobiodiversity of the Early Cretaceous (Berriasian) Angeac-Charente Lagerstätte (southwestern France): implications for continental faunal turnover at the J/K boundary. **Geodiversitas**, v. 44, n. 25, p. 683-752, 2022.

ANSORGE, J. Fischreste (Selachii, Actinopterygii) aus der Wealdentonscholle von Lobber Ort (Mönchgut/Rügen/DDR). **Paläontologische Zeitschrift**, v. 64, n. 1, p. 133-144, 1990.

BERMÚDEZ-ROCHAS, D. D. New hybodont shark assemblage from the Early Cretaceous of the Basque-Cantabrian Basin. **Geobios**, v. 42, n. 6, p. 675-686, 2009.

BHAT, M. S.; RAY, S.; DATTA, P. M. A new hybodont shark (Chondrichthyes, Elasmobranchii) from the Upper Triassic Tiki Formation of India with remarks on its dental histology and biostratigraphy. **Journal of Paleontology**, v. 92, n. 2, p. 221-239, 2018.

BLAKE, L. *et al*. Microvertebrates from the Lower Greensand Group (Lower Cretaceous) of Clophill, Bedfordshire, UK, and Nutfield, Surrey, UK. **Proceedings of the Geologists' Association**, v. 135, n. 5, p. 493-517, 2024.

BREEDEN III, B. T. *et al*. A new freshwater lonchidiid hybodontiform shark (Chondrichthyes, Elasmobranchii) from the Upper Triassic Momonoki Formation in Yamaguchi, Japan. **Journal of Vertebrate Paleontology**, p. e2322749, 2024.

DUFFIN, C. J. Revision of the hybodont selachian genus *Lissodus* Brough (1935). **Palaeontographica. Abteilung A, Paläozoologie, Stratigraphie**, v. 188, n. 4-6, p. 105-152, 1985.

DUFFIN, C. J.; THIES, D. Hybodont shark teeth from the Kimmeridgian (Late Jurassic) of northwest Germany. **Geologica et Palaeontologica**, v. 31, p. 235-256, 1997.

HECKERT, A. B.; IVANOV, A.; LUCAS, S. G. Dental morphology of the hybodontoid shark *Lonchidion humblei* Murry from the Upper Triassic Chinle Group, USA. New Mexico Museum of Natural History and Science Bulletin, v. 41 (**The Global Triassic**), p. 45–48, **Albuquerque: New Mexico Museum of Natural History and Science**, 2007.

KRIWET, J. Late Jurassic selachians (Chondrichthyes: Hybodontiformes, Neoselachii) from Central Portugal. **Neues Jahrbuch für Geologie und Paläontologie-Monatshefte**, p. 233-256, 2004.

KUMAR, J.; SHARMA, K. M. Study of the cynodont, mammal and other vertebrate fossils in the Tiki Formation, South Rewa Gondwana Basin, M. P., and study the faunal correlation with other part of the world. **Kolkata: Geological Survey of India (Government of India)**, 2013. v. 4.

MURRY, P. A. A new species of freshwater hybodont from the Dockum Group (Triassic) of Texas. **Journal of Paleontology**, p. 603-607, 1981.

ORESKA, M. P. J.; CARRANO, M. T.; DZIKIEWICZ, K. M. Vertebrate paleontology of the Cloverly Formation (Lower Cretaceous), I: faunal composition, biogeographic relationships, and sampling. **Journal of Vertebrate Paleontology**, v. 33, n. 2, p. 264-292, 2013.

PENN, S. J.; SWEETMAN, S. C. Microvertebrate-rich gutter casts from the basal Wessex Formation (Wealden Group, Lower Cretaceous) of Dungy Head, Dorset: Insights into the palaeoecology and palaeoenvironment of a non-marine wetland. **Cretaceous Research**, v. 143, p. 105397, 2023.

RAY, S. *et al*. Vertebrate fauna from the Late Triassic Tiki Formation of India: new finds and their biostratigraphic implications. **Journal of Palaeosciences**, v. 65, n. 1-2), p. 47-59, 2016.

REES, J. A new hybodont shark fauna from the Upper Jurassic Vitabäck Clays at Eriksdal, Scania, southern Sweden. **Dissertations in Geology at Lund University**, 1996.

REES, J. Shark fauna and depositional environment of the earliest Cretaceous Vitabäck Clays at Eriksdal, southern Sweden. **Earth and Environmental Science Transactions of The Royal Society of Edinburgh**, v. 93, n. 1, p. 59-71, 2002.

REES, J. Shark fauna and depositional environment of the earliest Cretaceous Vitabäck Clays at Eriksdal, southern Sweden. **Earth and Environmental Science Transactions of The Royal Society of Edinburgh**, v. 93, n. 1, p. 59-71, 2002.

REES, J.; UNDERWOOD, C. J. Hybodont sharks from the Middle Jurassic of the Inner Hebrides, Scotland. **Earth and Environmental Science Transactions of the Royal Society of Edinburgh**, v. 96, n. 4, p. 351-363, 2005.

REES, J.; UNDERWOOD, C. J. Hybodont sharks of the English Bathonian and Callovian (middle Jurassic). **Palaeontology**, v. 51, n. 1, p. 117-147, 2008.

REES, J. *et al*. Non-marine selachians from the basal Cretaceous of Charente, SW France. **Cretaceous Research**, v. 44, p. 122-131, 2013.

SHANG, Q.; CUNY, G.; CHEN, L. Early Middle Jurassic vertebrate microremains from the Three Gorges area, southern China. **Historical Biology**, v. 20, n. 2, p. 87- 99, 2008.

SINGH, N. A. *et al*. Enameloid ultrastructure of some chondrichthyan fishes (Hybodontiformes, Xenacanthiformes from the Late Triassic of Tiki Formation, Madhya pradesh; Carcharhiniformes and Myliobatiformes from Miocene of Kutch and Late Miocene Baripada beds, Orissa, India): an interrelationship and developmental approach. **Journal of the Palaeontological Society of India**, v. 65, n. 2, p. 115-130, 2020.

UNDERWOOD, C. J.; REES, J. Selachian faunas from the lowermost Cretaceous Purbeck Group of Dorset, southern England. In: **Life and Environments in Purbeck Times (Special Papers in Palaeontology)**. Paleontological Association, 2002. p. 83-101.

WEN, W. *et al*. Hybodontiform shark remains (Chondrichthyes, Elasmobranchii) from the Lower Triassic of Yunnan Province, China, with comments on hybodontiform diversity across the PTB. **Journal of Vertebrate Paleontology**, v. 42, n. 1, p. e2108712, 2022.
